# Development of Specific Markers for Identification of Biovars 1 and 2 Strains of *Pseudomonas syringae* pv. *actinidiae*

**DOI:** 10.5423/PPJ.NT.10.2015.0224

**Published:** 2016-04-01

**Authors:** Young Sun Lee, Gyoung Hee Kim, Young Jin Koh, Qiguo Zhuang, Jae Sung Jung

**Affiliations:** 1 Department of Biology, Sunchon National University, Sunchon 57922, Korea; 2 Department of Plant Medicine, Sunchon National University, Sunchon 57922, Korea; 3 Sichuan Provincial Academy of Natural Resources, Chengdu 610015, China

**Keywords:** bacterial canker, biovar, kiwifruit, molecular marker, *Pseudomonas syringae* pv. *actinidiae*

## Abstract

*Pseudomonas syringae* pv. *actinidiae*, the causal agent of canker in kiwifruit, can be divided into three biovars (biovars 1, 2, and 3). Strains belonging to biovar 1 produce phaseolotoxin and were isolated in Japan and Italy before 2008. Strains of biovar 2 produce coronatine instead of phaseolotoxin and have been isolated only in Korea. Strains belonging to biovar 3 produce neither phaseolotoxin nor coronatine and are responsible for the global outbreak of bacterial canker of kiwifruit in recent years. The biovar 3-specific primer set was developed in a previous work. In this study, two sets of PCR primers specific to strains of biovars 1 and 2, respectively, were developed based on random amplified polymorphic DNA analyses. Primers PsaJ-F and PsaJ-R produced a 481-bp region with genomic DNA of biovar 1 strains, whereas primers PsaK-F and PsaK-R amplified a 413-bp region present only in the genome of biovar 2 strains.

*Pseudomonas syringae* pv. *actinidiae* (hereafter called PSA) is the causal agent of bacterial canker of kiwifruit. This bacterium causes dark brown angular spots surrounded by yellow haloes, cankers with white or reddish exudation on twigs and trunks, wilting, and eventually plant mortality (Bastas and Karakaya, 2012; Koh et al., 2010; Vanneste et al., 2011). This pathogen was first described in Japan in 1989 (Takikawa et al., 1989) and was subsequently isolated in Korea (Koh et al., 1994) and Italy (Scotichini, 1994) in 1992. The Japanese and Korean populations of PSA have been reported to diverge evolutionarily. While Japanese strains produced phaseolotoxin, Korean strains synthesized coronatine as a phytotoxin (Han et al., 2003). Only a few reports are available on the situation of PSA in China where *Actinidia* plants are endemic species (Liang et al., 2000). In Italy, bacterial canker on kiwifruit caused by PSA was not a destructive disease when first reported in 1992 despite causing occasional leaf spot and twig dieback. However, PSA strain associated with more severe symptoms has been isolated from central Italy since 2008 (Ferrante and Scortichini, 2009). This strain has rapidly spread to other kiwifruit-producing regions in Italy and has been reported from other European countries, including Portugal (Balestra et al., 2010), Spain (Balestra et al., 2011), France (Vanneste et al., 2011), and Turkey (Bastas and Karakaya, 2012). In other continents, this pathogen has also been detected in New Zealand (Everett et al., 2011) and Chile (European Plant Protection Organization, 2011) in 2010. Bacterial canker caused by PSA has become the most serious limiting factor for cultivating kiwifruit worldwide.

The genetic relationships between PSA populations with different geographic origins have recently been investigated using various molecular methods. Ferrante and Scortichini (2010) used rep-PCR and multi-locus sequence analysis (MLSA) and found that PSA strains isolated in Italy during 2008–2009 are distinct from those previously reported in Japan and Italy. The unique characteristics of PSA populations isolated from recent outbreaks in Italy were confirmed by a random amplified polymorphic DNA (RAPD) analysis, which produced different profiles from those of previously isolated strains (Mazzaglia et al., 2011). Chapman et al. (2012) performed MLSA of housekeeping and type III effector genes in PSA strains from Japan, Korea, Italy, New Zealand, Australia, and Chile and demonstrated that at least four PSA MLSA groups, designated Psa 1–4 (biovar 1–4), are present globally. Biovar 1 includes strains isolated from Japan and Italy in 1992. Korean strains isolated since the late 1980s belong to biovar 2. Strains causing recent outbreaks in European countries, including Italy, New Zealand, and Chile, belong to biovar 3 (Mazzaglia et al., 2012). Strains belonging to biovar 3 are considered to cause more serious disease based on their aggressiveness and rapid spread (Scortichini et al., 2012; Young, 2012). Strains belonging to biovar 4 have been detected in New Zealand and Australia, apparently causing only leaf spots but no canker symptoms. Recently, biovar 4 has been shown to be a distinct and different pathovar of *P*. *syringae*, and it was assigned a new name of pv. *actinidifoliorum* (Cunty et al., 2015; Ferrante and Scortichini, 2015; McCann et al., 2013).

In Korea, biovar 2 strains have been isolated since the late 1980s causing severe economic losses to *A*. *deliciosa* cv. Hayward as well as *A*. *chinensis* cv. Hort16A (Koh et al., 1994, 2010). However, strains belonging to biovar 3 have also been isolated since 2011 (Koh et al., 2012). These strains display identical molecular characteristics with biovar 3 strains isolated from recent outbreaks (Koh, et al., 2014).

PCR primers have been developed to detect PSA or distinguish populations with different geographic origins. One of the primer pairs was designed within the 16S–23S rDNA intertranscribed spacer (ITS) region of PSA strains after comparison with other related species and pathovars (Rees-George et al., 2010). However, this primer set could not distinguish PSA from the phylogenetically similar pv. *theae*. Balestra et al. (2013) developed PCR primer sets based on genome sequences of PSA strains available from the GenBank database. They designed a primer pair to specifically detect PSA but not any *P*. *syringae* pathovar, including pv. *theae*, as well as three additional primer sets to distinguish PSA populations originating from Europe, China, and Japan/Korea. However, the Japan/Korea primer set could not distinguish biovar 1 from biovar 2.

In this work, biovar 1 and 2-specific primer sets were developed based on RAPD analyses. Twelve PSA strains, including four biovar 1, three biovar 2, and five biovar 3 strains, were used for RAPD analyses and for primer validation (Table 1). Bacterial strains were cultured at 28°C in peptone-sucrose (20 g of peptone, 20 g of sucrose per 1 liter) medium. Total DNA was isolated from bacteria using an AccuPrep genomic DNA extraction kit (Bioneer, Korea).

The existence of genes related to phytotoxin production were tested in order to confirm that the four strains isolated in Japan belong to biovar 1 and the three strains isolated in Korea belong to biovar 2. While biovars 1 and 2 each produce phaseolotoxin and coronatine, respectively, biovar 3 produces neither (Ferrante and Scortichini, 2010; Han et al., 2003). PCR amplification procedures have been applied to detect coronatine- or phaseolotoxin-producing *P*. *syringae* pathovars. The PCR primer set OCT-F/R, based on the *argK* sequence encoding phaseolotoxin-resistant ornithine carbamoyltransferase, has been used for specific detection of phaseolotoxin-producing *P*. *syringae* pathovars (Sawada et al., 1997). The PCR primer set CFL-1/2, derived from *cfl* gene encoding coronafacate ligase, has been used to detect coronatine-producing *P*. *syringae* strains (Bereswill et al., 1994). As shown in Fig. 1, the OCT-F/R primer set amplified a 1,098-bp fragment with four strains isolated from Japan and Italy in 1992. Three strains isolated in Korea amplified an expected 655-bp fragment using the CFL-1/2 primers. However, no PCR products were produced when these primer sets were applied to biovar 3 strains.

The first primer set was designed to specifically detect biovar 1 strains, but not biovar 2 or biovar 3 strains. RAPD amplification was carried out throughout this work in 50-μl reaction mixtures containing 30 ng of genomic DNA, 5 μl of 10× reaction buffer, 5 μl of 10 mM dNTP mix, 20 pmol of single primer, and 2.5 U of *Top* DNA polymerase (Bioneer, Korea). The random primer OPA-2 (Operon Biotechnologies, USA) amplified a distinct band of approximately 650 bp present in four biovar 1 strains (data not shown). The differential RAPD band was excised from 2.0% agarose gel, and the DNA was purified using an AccuPrep gel purification kit (Bioneer, Korea). The DNA eluted from the band was ligated into pGEM-T Easy vector (Promega, USA) and then transformed into competent *Escherichia coli* JM109. The inserted DNA fragment was sequenced by SolGent Co. (Korea). Nucleotide sequence revealed that the exact nucleotide length of the specific amplicon was 641 bp, including 10-mer random primers at each end. This sequence showed 93% homology with the PPHGI-1 genomic island sequence of *P*. *syringae* pv. *phaseolicola* retrieved from the National Center for Biotechnology (NCBI) database using the BLAST algorithm. The amplified region is located within the nucleotide sequence of the genomic island between base pairs 16717 and 17353 of GenBank accession number AJ870974.1. The biovar 1-specific primer set PsaJ-F/R was designed using primer3 software (Untergasser et al., 2012) based on internal sequences of the RAPD marker. This primer set amplified 481-bp fragment only from DNA of biovar 1 strains of PSA (Fig. 2A).

The same methods used to design biovar 1 were applied to develop biovar 2-specific primers. The random 10-mer primer OPA-16 produced an approximately 450-bp biovar 2-specific band. Sequencing showed that the size of this fragment was 462 bp but did not detect any significant homology to known sequences available from the NCBI database. PCR primers PsaK-F/R were designed from the nucleotide sequences of the biovar 2-specific RAPD marker. This primer pair amplified a 413-bp region present only in the genome of biovar 2 of PSA (Fig. 2B). The nucleotide sequences, annealing temperatures, and amplicon sizes of the primers used in this work are listed in Table 2.

Amplifications were carried out in a DNA Thermal Cycler (Takara Shozo, Japan) under the following conditions: an initial denaturation at 95°C for 5 min, followed by 30 cycles of a denature step of 95°C for 30 s, annealing for 30 s, extension at 72°C for 30 s, and final extension at 72°C for 7 min.

Two primer sets, PsaJ-F/R and PsaK-F/R, were validated with an additional five biovar 1 and twenty biovar 2 strains of PSA. The 481-bp biovar 1 fragment and 413-bp biovar 2 amplicon were produced with corresponding primer sets and DNA from PSA strains isolated from Japan and Korea, respectively (data not shown). In order to verify the specificity of the designed primer sets, both primer sets were tested on 15 bacterial strains, including 10 *P*. *syringae* pathovars; *P*. *syringae* pv. *glycinea*, pv. *syringae*, pv. *tabaci*, pv. *viridiflava*, pv. *maculicola*, pv. *tomato*, pv. *atropurpurea*, pv. *phaseolicola*, pv. *morsprunorum* and pv. *theae*, and five plant pathogenic bacteria; *P*. *fluorescens*, *P*. *veronii*, *P*. *rhodesiae*, *Xanthomonas campestri* pv. *pruni*, and *Acidovorax valerianellae*. No signal was obtained from these 15 bacterial strains when using the two primer sets, confirming the reliability of these primers in discriminating between biovar 1 and 2 strains of PSA (Fig. 3A, B). However, when we performed PCR with OCT-F/R primer set, 1,098-bp fragments were amplified from not only biovar 1 strain but also *P*. *syringae* pv. *phaseolicola* and pv. *tabaci* (Fig. 3C). In the case of PCR performed with CFL-1/2 primer set, the expected fragments of 655 bp were amplified from *P*. *syringae* pv. *glycinea* and pv. *atropurpurea* together with the biovar 2 strain of PSA (Fig. 3D).

These biovar 1 and 2-specific primers can be used to determine the biovar group of PSA strains together with biovar 3-specific primers, which were reported in the previous study (Koh et al., 2014). The PCR assay developed in this study may serve as a useful tool for identification of biovars of PSA and will consequently be helpful in monitoring the migration of PSA strains.

## Figures and Tables

**Fig. 1:**
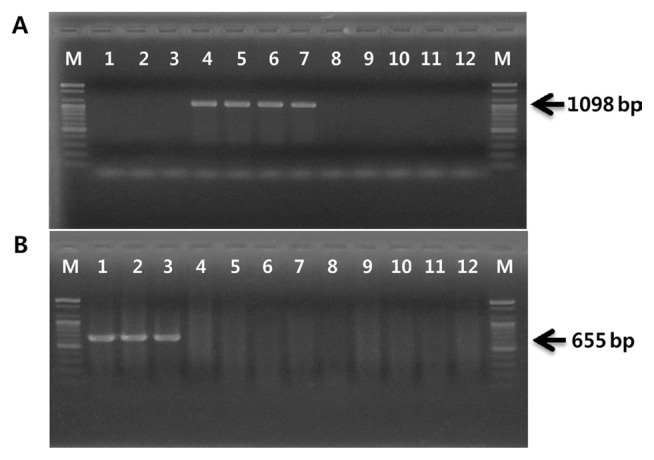
PCR amplification products obtained with DNA of *Pseudomonas syringae* pv. *actinidiae* strains and primers, OCT-F/R (A) and CFL-1/2 (B). Lane M, 100-bp marker (Bioneer); lane 1, CJW7; lane 2, KBE9; lane 3, KBE29; lane 4, KW11; lane 5, SUPP 319; lane 6, SUPP 320; lane 7, NCPPB 3871; lane 8, SYS1; lane 9, P1; lane 10, 155; lane 11, CFBP 7286; lane 12, ICMP 18708.

**Fig. 2:**
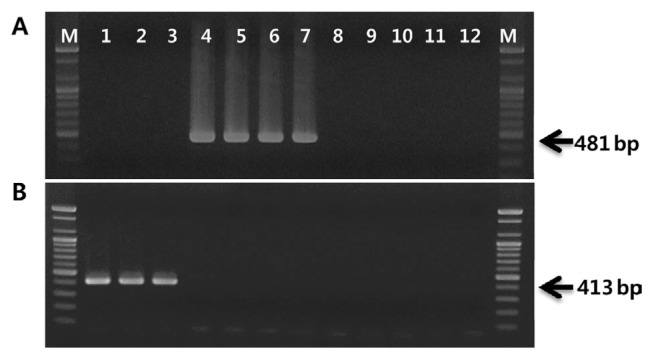
Agarose gel showing PCR amplification products using biovar 1-specific primer pair, PsaJ-F/R (A) and biovar 2-specific primer pair, PsaK-F/R (B). Lanes are the same as Fig. 1.

**Fig. 3:**
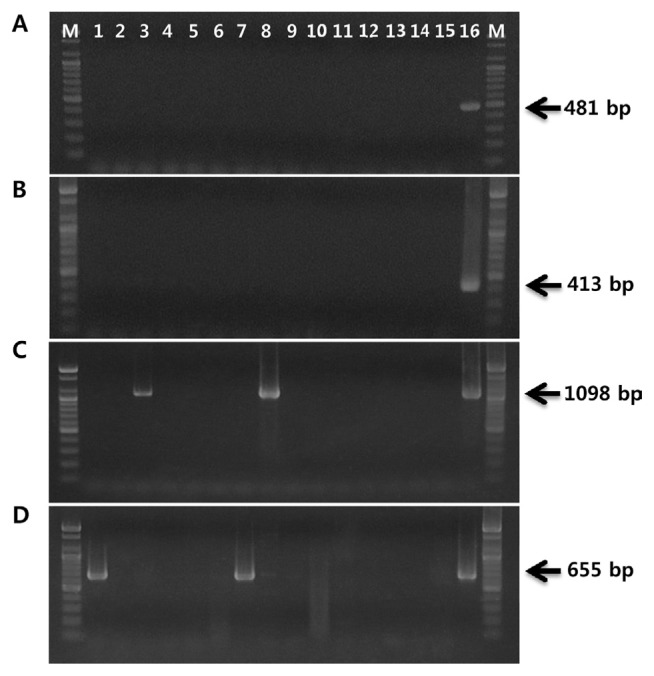
Specificity of PCR assay using biovar 1 primer pair, PsaJ-F/R (A), biovar 2 primer pair, PsaK-F/R (B), OCT-F/R (C), and CFL-1/2 (D). Ten *Pseudomonas syringae* pathovars and five plant pathogenic bacteria were used to test specificity of the designed primer pairs. Lane M, 100-bp marker (Bioneer); lane 1, *Pseudomonas syringae* pv. *glycinea*; lane 2, *P. s*. pv. *syringae*; lane 3, *P. s.* pv. *tabaci*; lane 4, *P. s.* pv. *viridiflava*; lane 5, *P. s.* pv. *maculicola*; lane 6, *P. s.* pv. *tomato*; lane 7, *P. s.* pv. *atropurpurea*; lane 8, *P. s.* pv. *phaseolicola*; lane 9, *P. s.* pv. *morsprunorum*; lane 10, *P. s.* pv. *theae*; lane 11, *P. fluorescens*; lane 12, *P. veronii*; lane 13, *P. rhodesiae*; lane 14, *Acidovorax valerianellae*; lane 15, *Xanthomonas campestri* pv. *pruni*; lane 16, *P. syringae* pv. *actinidiae* KW11 (A, C) or *P. syringae* pv. *actinidiae* CJW7 (B, D).

**Table 1:** Bacterial strains used in this work

*Pseudomonas syringae* pv. *actinidiae*			

Strain	Isolated country	Year	Biovar
CJW7	Korea	1999	2
KBE9	Korea	2008	2
KBE29	Korea	2011	2
KW11	Japan	1984	1
SUPP 319	Japan	–	1
SUPP 320	Japan	–	1
NCPPB 3871	Italy	1992	1
SYS1	Korea	2011	3
P1	China	2014	3
155	China	2014	3
CFBP 7286	Italy	2011	3
ICMP 18708	New Zealand	2010	3

Bacterial strains used for specificity test of primers			

*Pseudomonas syringae* pv. *glycinea* ATCC 8727		*P*. *s*. pv. *syringae* DSM 10604	
*P*. *s*. pv. *tabaci* DSM 1856		*P*. *s*. pv. *viridiflava* LMG 2352	
*P*. *s*. pv. *maculicola* LMG 5071		*P*. *s*. pv. *tomato* DSM 50315	
*P*. *s*. pv. *atropurpurea* DSM 50255		*P*. *s*. pv. *phaseolicola* NPS 3121	
*P*. *s*. pv. *morsprunorum* DSM 50277		*P*. *s*. pv. *theae* LMG 5093	
*P*. *fluorescens* LMG 1794		*P*. *veronii* KACC 10802	
*P*. *rhodesiae* KACC 10811		*Acidovorax valerianellae* SMBL4	
*Xanthomonas campestri* pv. *pruni* MAFF 301421			

**Table 2:** List of primers used in this work

Primer Name	Sequence (5′-3′)	Annealing Temp. (°C)	Size (bp)	Target	Reference
OPA-2	TGCCGAGCG	37	-	random primer	-

OPA-16	AGCCAGCGAA	37	-	random primer	-

OCT-F	TATTACCCTGATGAGCTCGA	58	1,098	*argK*	Sawada et al., 1997
OCT-R	GATGATCGACCTTGTTGACCTCCCG				

CFL-1	GGCGCTCCCTCGCACTT	65	655	*cfl*	Bereswill et al., 1994
CFL-2	GGTATTGGCGGGGGTGC				

PsaJ-F	GACGTCGACGACAAGGTGAT	65	481	biovar 1	This study
PsaJ-R	AGTAAACCGTGCCGTCATCTC				

PsaK-F	GACAAAGCCAAAAAGGCGA	65	413	biovar 2	This study
PsaK-R	TGCCAAGCCCAAGTATCCAAGC				
